# A novel method of using Deep Belief Networks and genetic perturbation data to search for yeast signaling pathways

**DOI:** 10.1371/journal.pone.0203871

**Published:** 2018-09-12

**Authors:** Songjian Lu, Xiaonan Fan, Lujia Chen, Xinghua Lu

**Affiliations:** 1 Department of Biomedical Informatics, University of Pittsburgh, Pittsburgh, Pennsylvania, United States of America; 2 Department of Automation, Northwestern Polytechnical University, Shanxi, People’s Republic of China; King's College London, UNITED KINGDOM

## Abstract

Perturbing a signaling system with a serial of single gene deletions and then observing corresponding expression changes in model organisms, such as yeast, is an important and widely used experimental technique for studying signaling pathways. People have developed different computational methods to analyze the perturbation data from gene deletion experiments for exploring the signaling pathways. The most popular methods/techniques include K-means clustering and hierarchical clustering techniques, or combining the expression data with knowledge, such as protein-protein interactions (PPIs) or gene ontology (GO), to search for new pathways. However, these methods neither consider nor fully utilize the intrinsic relation between the perturbation of a pathway and expression changes of genes regulated by the pathway, which served as the main motivation for developing a new computational method in this study. In our new model, we first find gene transcriptomic modules such that genes in each module are highly likely to be regulated by a common signal. We then use the expression status of those modules as readouts of pathway perturbations to search for up-stream pathways. Systematic evaluation, such as through gene ontology enrichment analysis, has provided evidence that genes in each transcriptomic module are highly likely to be regulated by a common signal. The PPI density analysis and literature search revealed that our new perturbation modules are functionally coherent. For example, the literature search revealed that 9 genes in one of our perturbation module are related to cell cycle and all 10 genes in another perturbation module are related by DNA damage, with much evidence from the literature coming from *in vitro* or/and *in vivo* verifications. Hence, utilizing the intrinsic relation between the perturbation of a pathway and the expression changes of genes regulated by the pathway is a useful method of searching for signaling pathways using genetic perturbation data. This model would also be suitable for analyzing drug experiment data, such as the CMap data, for finding drugs that perturb the same pathways.

## Background

Understanding cellular signaling pathway systems is one of the major tasks those in the systems biology field undertake [1]. Many important cell activities, such as proliferation and apoptosis, can be regulated by signaling pathways that accept signals from the surface of cells, where the pathways regulate cell activities by adjusting the expression levels of corresponding down-stream genes. Hence, the study of pathways can help us to understand the mechanism of diseases, such as cancer, that are caused by genetic problems [2, 3].

One well established technology that can be used to study the cell signaling system is genetic perturbation experiments, i.e., observing cell expression profile changes by deleting protein-coding genes in model organisms, such as yeast. For example, Hughes et al. performed a pioneering study of yeast (*Saccharomyces cerevisiae*) signaling systems by generating and studying genome-wide mRNA expression profiles with the deletion of 276 protein-coding genes [4]. Very recently, Kemmeren et al. generated a new data set with the mRNA expression profiles of 1484 deletion mutations of protein-coding genes for the study of yeast regulatory systems [5]. This type of experiment has generated a large amount of expression data [4–6] that provides opportunities for studying the signaling system using computational methods.

One group of popular computational methods is clustering based, such as the hierarchical or k-means clustering. The basic idea is that if two genes have similar expression profiles across all samples or the deletions of two genes have similar genome-wide expression profiles, then these two genes are functionally related. Kemmeren et al. used hierarchical clustering to study the expression data and found that if genes are in the sample protein complex or the same pathway, then genome-wide expression profiles of deletions of these genes were significantly similar [5]. There are some other works [7–11] that combined expression data with other knowledge or techniques to search for or study signaling pathways. For example, Steffen et al. combined gene expression data, protein-protein interaction network, and k-mean algorithm to search for sub-network [9]. Their basic idea was that genes in a sub-network were more likely to belong to a pathway if they were in one cluster obtained from clustering the expression data. Zhao et al. applied expression profile and mutual exclusivity to find pathways related to cancer development [10]. They thought that if mutations of genes were mutually exclusive among tumors, and furthermore, gene expressions of those genes were also similar across all tumors, then those genes were likely to be on the same pathway. In a summary, the purpose of using expression data in previous works was similar, i.e., genes in a pathway should have similar expression profiles across all samples or expressed genes.

Our major motivation for proposing a new computational model to search for signaling pathways in this work is that (to our best knowledge) previous methods did not consider or not fully utilize the intrinsic relation between the perturbation of a pathway and expression changes of genes regulated by the pathway. It is obvious that if a deletion perturbs a pathway, i.e., a gene/protein in a pathway has been deleted, then expression levels of genes regulated by the pathway should change significantly. However, when we measure those expression changes, certain random perturbations, such as the variance of the microarray products and the impact of artificial factors in the experiments, are hard to avoid. For example, researchers may have noticed expression differences of genes among wild-type or control samples. People may also have found that in many data sets, even the deletion of the same gene in two samples under the same condition, the significantly changed genes in the two samples were quite different. So in the expression data resulted from the deletion of a gene in a pathway, besides the genes regulated by the pathway, some other random genes may also be differentially expressed. If we used genome-wide expression profiles to study the relations between deleted genes using some traditional methods, such as the hierarchical or k-means clustering, those random perturbations would cause problems as expression of a large number of genes not regulated by the pathway also make contribution in those clustering methods. In this project, in order to reduce above problems, we first search for gene modules, called transcriptomic modules, such that genes in each transcriptomic module are highly likely to be regulated by a common pathway. We then use the expression status of each transcriptomic module as a readout of pathway perturbations to search for an up-stream perturbation module such that genes in the perturbation module come from the same pathway. *The random perturbations should be greatly reduced if we only consider the expression changes of genes regulated by each pathway respectively when we search for up-stream perturbation modules*.

We use a deep learning technique called deep belief network (DNB) [12, 13] to search for transcriptomic modules as it can learn the hierarchical structure that exists within the differentially expressed genes of the perturbation data. The DBN is a machine learning technique that was originally developed for image processing, such as face recognition. A DBN may have one visible layer and multiple hidden layers. When a DBN is applied for face recognition, its nodes in the first, second, and third hidden layers can group pixels that make edges (line, curve segments, etc.), components (eye, nose, mouse etc.), and faces together, respectively [14, 15], i.e., it can learn the hierarchical structure that exists within the input data. In the cascade structure of a pathway system, a gene/protein in up-stream usually controls more genes (gene expressions) than a gene in down-stream does. However genes controlled by different genes along the same pathway should have a hierarchical structure. We use DBN to learn this hierarchical structure and to search for down-stream transcriptomic modules such that genes in each transcriptomic module are commonly regulated by one signaling pathway. The major difference of using DBN and other clustering methods, such as hierarchical clustering or k-means method, is that the DBN is finding gene modules such that genes in each module are co-differentially expressed in a number of samples that are statistically significant while the hierarchical clustering and k-means method are searching for genes with similar expression values in all samples. So for the hierarchical clustering and k-means method, expression values of genes in samples that do not have the significant expression changes of those genes also affect the clustering results. As the lengths of pathways are usually not very long, in the perturbation data set, genes regulated by each signaling pathway should not be co-differentially expressed in many samples. Using DBN is a better way to find genes regulated by each pathway as genes regulated by each pathway were only co-differentially expressed in a very small number of perturbed samples.

Our paper is organized as follows. After the introduction section, we introduce the methods of our model in detail. We then introduce the results, including evaluation. Finally, we present our conclusions.

## Methods

### Data collection and preprocessing

We collected the gene expression data of 1484 samples [5], where each sample is the mRNA expression profile of the deletion of one protein-coding gene in *Saccharomyces cerevisiae*. Each profile includes expression level in the form of standard deviation, average transcription level changes (fold changes) in the mutant relative to 428 WTs, and *p*-values. In the preprocessing step, we used the setting of the paper [5] to find what genes were differently expressed under the deletion, i.e. a gene was considered to be differently expressed if its fold change was at least 1.7 and the *p*-value was less than 0.05. After the preprocessing, we obtained a 0/1 matrix such that each row is for a measured gene and each column for a sample (perturbed gene); a value 1 represented that a gene was differently expressed in a sample; otherwise the value was set to 0. This 0/1 matrix was used to train the Deep Belief Network (DBN). The 0/1 matrix depends on the threshold setting, which may lead to the change of trained DBN. However, if a method is stable, the results should not change too much for a little change of threshold setting. We have tested to use a fold change cutoff of 1.75 and 1.65 to make 0/1 matrices, respectively. We found that the results of DBN from 0/1 matrices of different fold change cutoffs were very similar, which provides evidence that the DBN model is quite s. Note: when the DBN program loads this 0/1 matrix, the number of nodes in the visible layer is set as the number of measured genes in the 0/1 matrix.

### Training the DBN with the 0/1 matrix

**Fig 1:**
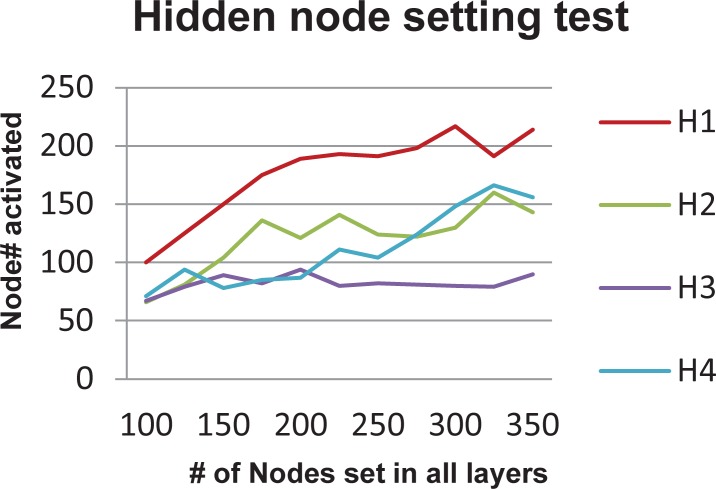
Numbers of hidden nodes that were set and actually activated in all hidden layers.

In the introduction, we stated that differentially expressed genes caused from the perturbations of different locations of a signaling pathway have a hierarchy structure that can be discovered by a DBN. People usually use three or four hidden layers for DBN, where the default number of hidden layers is four in the Matlab codes provided by Hinton et al. In this work, we used a DBN with four hidden layers to learn this hierarchy structure, where the number of nodes in the visible layer is the number of measured genes (row number of the 0/1 matrix), and the number of nodes in the first, second, third and fourth hidden layers are 217, 160, 94, and 166 respectively. To obtain a good performance, setting a proper number of nodes in each hidden layer is important. The number of nodes needed in each hidden layer depends on the input training data. As there is no “gold standard”, people usually adjust those parameters manually. In this project, we introduce a better way to estimate the number of nodes needed in each hidden layer. After the DBN is trained by a given input data, for each node in any hidden layer and each sample in the input data, the DBN returns the probability that the hidden node is activated in the sample. Hence, we can know how many nodes in each hidden layer have been activated with a probability of *p* in at least one sample. We found that for a fixed probability *p*, such as 0.75, if we set the number of nodes in all hidden layers to *k* and gradually increased this *k*, the number of nodes that were activated with probability *p* in at least one sample in each layer fluctuated around a certain number (refer to Fig 1). For example, if we set *k* to be 100, 125, 150, 175, 200, 225, 250, 275, 300, 325, and 350, then the number of nodes that were activated with a probability of 0.75 at the first hidden layer would be 100, 125, 150, 175, 189, 193, 191, 198, 217, 191, and 214 respectively. We found that the DNB was able to achieve good performance if we found the maximum number obtained from the different settings of *k* for each hidden layer and then used this maximum number to set this hidden layer, for example, in the previous case, setting the number of nodes in the first hidden layer to be 217.

### Obtaining the transcriptomic modules

After the DBN was trained, for each node *T* in the first hidden layer, we learned the weights for all edges from the node *T* to all nodes (genes) in the visible layer. Each edge weight represents how strongly the value of the visible layer is affected by the value of node *T*. We computed the mean *μ* and standard deviation *δ* from all the weights of the edges from *T* to all of the nodes in the visible layer. Then we used *μ* and *δ* to set a threshold, a *p*-value of 0.05, for choosing a set of nodes (genes) *S* in the visible layer and considered them to be regulated by the node *T*. We considered genes in *S* to be a transcriptomic module.

### Searching for up-stream perturbation modules

For each transcriptomic module, we tried to find a set of genes that was highly likely to be on the pathway regulating the expression of the transcriptomic module. We called this set of genes a perturbation module, as the deletion of any gene in the perturbation module would perturb the expression of the corresponding transcriptomic module. We only considered genes that were deleted in the data set we collected. For each down-steam transcriptomic module, we **first** found a gene, *called the initial gene*, such that the deletion of the initial gene caused the most number of genes in the transcriptomic module to be differently expressed. We **then** iteratively added new genes into the solution such that every time a new gene was added, it would have the shortest average distance to all of the other genes in the previous solution. For a transcriptomic module, the distance between any two genes *G*_1_ and *G*_2_ was decided by the *normalized* expression level changes (normalized fold changes) of the genes in the transcriptomic module with the deletions of *G*_1_ or *G*_2_, where the normalized fold change is defined as:

$$norm(x)=\left\{ \begin{array}{l} \;2,\;x\geq 1.7\;\mathrm{and}\;p_{\_\mathrm{Value}}<0.05\\ \;1.7,\;x\geq 1.7\;\mathrm{and}\;p_{\_\mathrm{Value}}\geq 0.05\\ \;x,\;-1.7<x<1.7\\ -1.7,\;x\leq -1.7\;\mathrm{and}\;p_{\_\mathrm{Value}}\geq 0.05\\ \;-2,\;x\leq -1.7\;\mathrm{and}\;p_{\_\mathrm{Value}}<0.05 \end{array}.\right.$$

We normalized the fold changes of the gene expression levels as we wanted the fold change on a gene not to contribute to the distance if it is up- or down-regulated significantly in both gene deletions. As in the original data, the expression of a gene was considered to change significantly if the fold change was at least 1.7 and the *p*-value was less than 0.05, so we wanted to distinguish cases of *p*-value ≥ 0.05 and *p*-value<0.05. When the fold change is at least 1.7, we set the value to be ±2 when the *p*-value<0.05. Specifically, suppose that the transcriptomic module has genes *g*_1_, *g*_2_, …, *g*_*t*_; the deletion of *G*_1_ would cause the expression changes of those *t* genes to be *u*_1_, *u*_2_, …, *u*_*t*_, and the deletion of *G*_2_ would cause the expression change of those *t* genes to be *v*_1_, *v*_2_, …, *v*_*t*_; then the distance of *G*_1_ and *G*_2_ would be the minimum Euclidean distances between vectors (*u*_1_, *u*_2_, …, *u*_*t*_) and (*v*_1_, *v*_2_, …, *v*_*t*_) and the Euclidean distances between vectors (*u*_1_, *u*_2_, …, *u*_*t*_) and -(*v*_1_, *v*_2_, …, *v*_*t*_). We consider the Euclidean distances between vectors (*u*_1_, *u*_2_, …, *u*_*t*_) and -(*v*_1_, *v*_2_, …, *v*_*t*_) as the deletion of a gene on the pathway may inhibit the signal while the deletion of another gene on the pathway may enhance the signal. Therefore, the expression changes of genes regulated by a pathway may be in the reverse direction for the deletion of different genes on the same pathway.

## Results

We obtained 217 transcriptomic modules. Correspondingly, we found 217 up-stream perturbation modules. In this section, we first give a systematic evaluation of the transcriptomic and perturbation modules. We then present more detail of some of our perturbation modules.

In our hypothesis, genes in a transcriptomic module are highly likely to be regulated by a pathway or even by a transcription factor. Hence, to evaluate our transcriptomic modules, we first determined whether genes in a transcriptomic module were enriched in genes regulated by known transcription factors. Then we investigated whether the genes in the transcriptomic modules were functionally coherent.

### Verifying transcriptomic modules with transcription factors

In the gene expression data that we collected, there existed deletions of 67 transcription factors that caused at least 10 genes to be differently expressed. As a result, we could obtain genes that are regulated by those 67 transcription factors. Using enrichment analysis, we first checked the overlap of genes in the transcriptomic modules with genes regulated by those 67 transcription factors. Remember that genes in each transcriptomic module are regulated by a node in the first hidden layer. After the DBN was trained using given training data, we obtained information about the probability that a node in the first hidden layer would be activated in each sample in the training data. We chose the 0.95 as the confidence threshold to decide if a node in the first hidden layer would be activated in a sample. Hence, we obtained information about how many times a node in the first hidden layer was activated in the training data.

**Fig 2:**
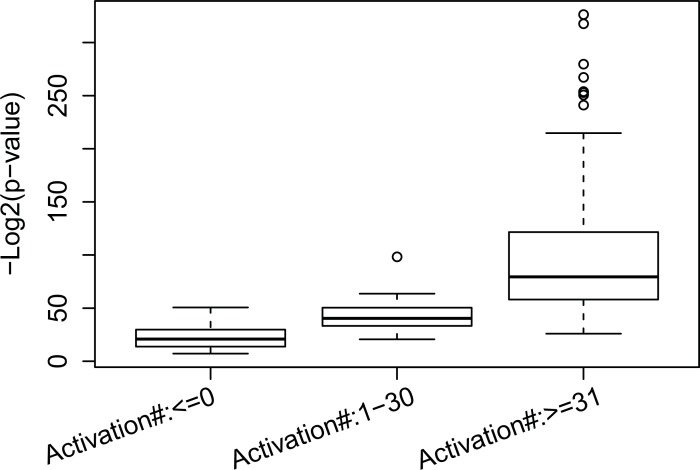
Comparing the TF enrichment analysis of genes regulated by the first layer hidden nodes with different activation numbers.

By intuition, we know that if a node *h* is activated in only a very few or even no samples, then the weights from the node *h* to all nodes in the visible layer will not be well trained. Therefore, genes in transcriptomic modules regulated by the node *h* should be less reliable than genes in transcriptomic modules regulated by a node that is activated many times. Our results supported this hypothesis. We split our transcriptomic modules into three groups according to the number of activations of their corresponding nodes in the first hidden layer, i.e. modules in group 1, 2, and 3 are regulated by nodes that are activated 0, between 1 and 30, and more than 30 times, respectively. Our results show that the average enrichment *p*-values (negative log value with base 2) for transcriptomic modules in group 1, 2, and 3 were 22.03, 42.21, and 101.2, respectively (refer to Fig 2). Looking back to the original space, on average, the enrichment *p*-values for transcriptomic modules in group 2 were 9.8×10^5^ fold better than those for transcriptomic modules in group 1, and the enrichment *p*-values for transcriptomic modules in group 3 were 5.7×10^17^ fold better than those for transcriptomic modules in group 2.

### Verifying transcriptomic modules using Gene Ontology (GO)

**Fig 3:**
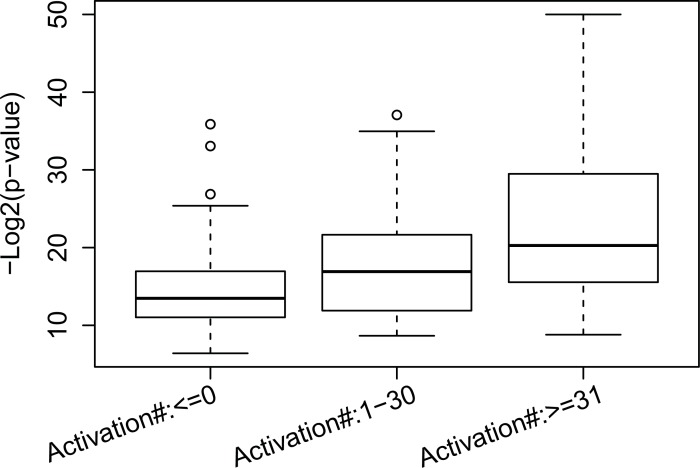
Comparing the GO enrichment analysis of genes regulated by the first layer hidden nodes with different activation numbers.

We also verified our transcriptomic modules using Gene Ontology to determine whether the genes in each transcriptomic modules were functionally coherent. As we supposed that the genes in each transcriptomic module are regulated by a signaling pathway, they should be functionally coherent. For each transcriptomic module, we searched for a GO term such that genes in the transcriptomic modules were most enriched in the GO term, i.e. had the minimum *p*-value for the hypergeometric test. We still compared the enrichment *p*-values (negative log value with base 2) for the transcriptomic modules in the three groups above. These results also showed that the transcriptomic modules in group 3 had the best enrichment *p*-values, where the average *p*-values (in log space) for the transcriptomic modules in group 1, 2, and 3 are 14.38, 17.36, and 24.02, respectively (refer to Fig 3). A global enrichment analysis of top 20 most enriched GO terms and their matched transcriptomic modules can be found at S1 Fig The result shows that many top 20 enriched GO terms are related to metabolic process and some transcriptomic modules can be significantly enriched in more than one GO term.

### Verifying perturbation modules using protein-protein interactions (PPIs)

**Fig 4:**
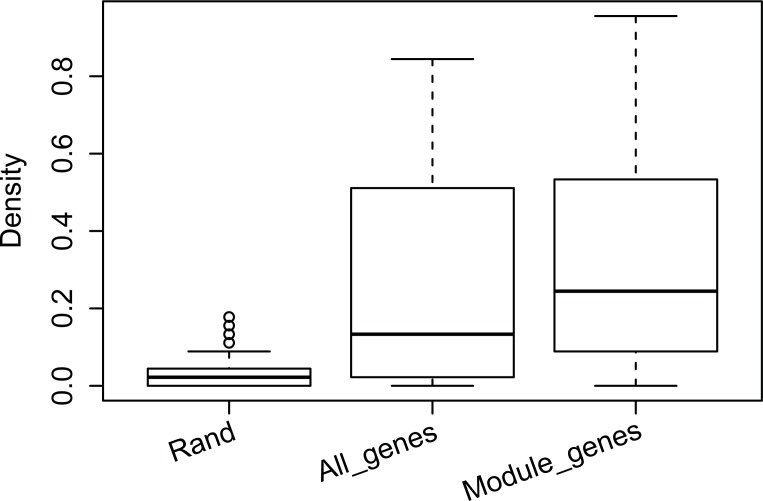
Comparing the PPI density of genes in perturbation modules obtained from different methods.

According to our hypothesis, the genes in each perturbation module are highly likely to be on the same signaling pathway. As PPIs are important for signal transduction [16, 17], we expected that there would be more PPIs among genes in our perturbation modules than among genes that are obtained using a random process. As the lengths of signaling pathways are usually not very long, the sizes of perturbation modules should not be too large. To test our model, we fixed the size of the perturbation modules to 10. The results agree with our expectation (refer to Fig 4). We found that the average PPI density, i.e. the ratio of the actual PPI number and total number all gene pairs, for 10 genes randomly chosen from the deleted genes in the expression data set was 0.034 (marked as “Rand”) while the average density for genes from our perturbation module was 0.33 (Marked as “Module_genes”). If we use the same algorithm for finding our perturbation modules, but instead of constraining genes in the transcriptomic modules we use all genes to search for 10 genes, the average PPI density is only 0.27. Hence, by using only the genes in the transcriptomic modules to search for perturbation modules, we can greatly improve performance in terms of PPI density.

### Verifying perturbation modules using Gene Ontology (GO)

**Fig 5:**
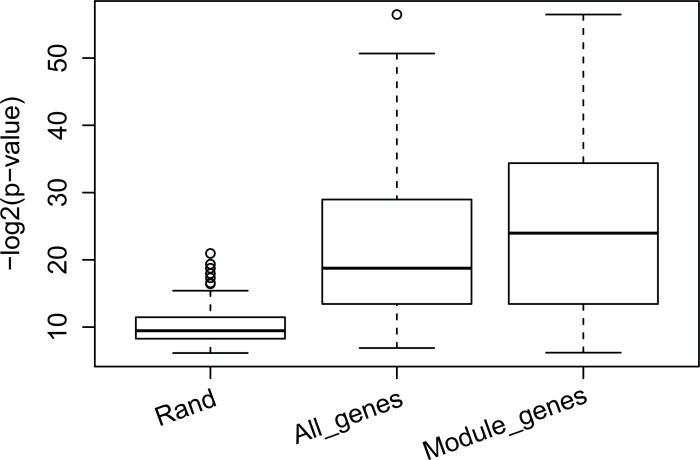
Comparing the GO enrichment analysis of genes in perturbation modules obtained using different methods.

We also verified our perturbation modules using Gene Ontology and found that results of the analysis of our perturbation was similar to those of the analysis using PPI density. The average enrichment *p*-values (negative log value with base 2) of random perturbation modules was 9.48 while the average enrichment p-values of our perturbation modules was 25.50, where the difference was 6.65×10^4^ in the original space. In the sample time, the average enrichment for perturbation modules obtained from all genes was 18.76 (refer to Fig 5). Hence, the GO analysis also proved that transcriptomic modules could be greatly helpful in finding perturbation modules. A global enrichment analysis of top 20 most enriched GO terms and their matched perturbation modules can be found at S2 Fig GO enrichment analysis also shows that the normalization of fold change also improve the performance of the perturbation module finding (refer to S3 Fig).

## Literature search revealed that genes in our perturbation modules are functionally coherent

**Fig 6:**
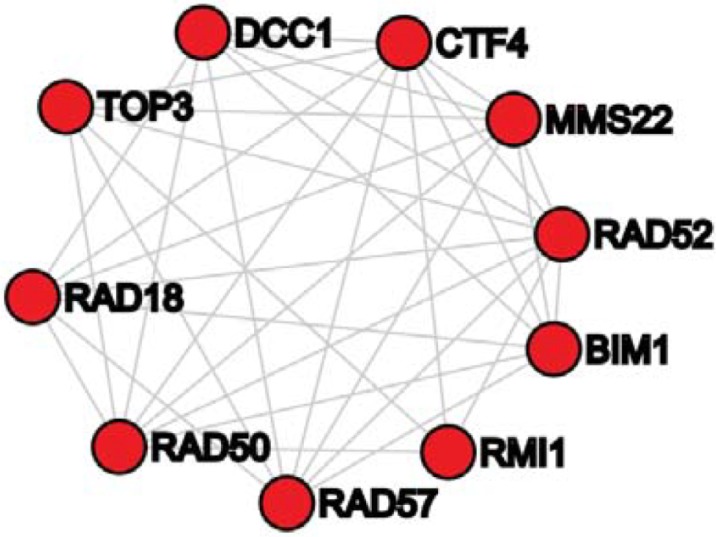
Protein-protein interaction subnetwork of genes in the perturbation module-30.

We conducted a literature search to study genes in our perturbation modules and found that the genes in our perturbation modules are functionally coherent. As the literature search has to be done manually, it is hard to verify many modules. In this section, we only report the results of literature search for two perturbation modules. One perturbation module, denoted as Module-30, has genes BIM1, JNM1, MMS22, NPL3, RAD18, RAD50, RAD52, RMI1, SGS1, and TOP3. There exist many protein-protein interactions among these10 genes (refer to Fig 6). We found that 9 of these 10 genes are associated with the cell cycle. Voncken et al. found that BIM1 is cell cycle-regulated and associated with the G(1)-phase of the cell cycle [18]. McMillan [19] and Wang [20] et al. revealed that JNM1 regulates the spindle orientation during the mitotic cell cycle. Vaisica et al. showed that the deletion of MMS22 caused an abnormal cell cycle [21]. Dovey et al. also verified that the loss of MMS22 had an impact on the S- and G2-phases of the cell cycle [22]. Bi et al. showed that RAD18 regulates the recovery from S-phase checkpoint-mediated arrest [23]. Zhu [24] and Gatei et al. [25] found that RAD50 is related to cell cycle regulation. Lisby discovered that RAD50 is associated with the DNA repair and recombination centers during the S-phase of the cell cycle [26]. Xu et al. presented that RMI1 is related to the M-phase of the cell cycle [27]. Balogun et al. found that the loss of SGS1 significantly impairs activation of cell cycle arrest [28]. Mankouri et al. showed that TOP3 is required for normal S-phase progression after DNA damage [29]. Therefore, it is highly likely that genes in Module-30 regulate the cell cycle.

**Fig 7:**
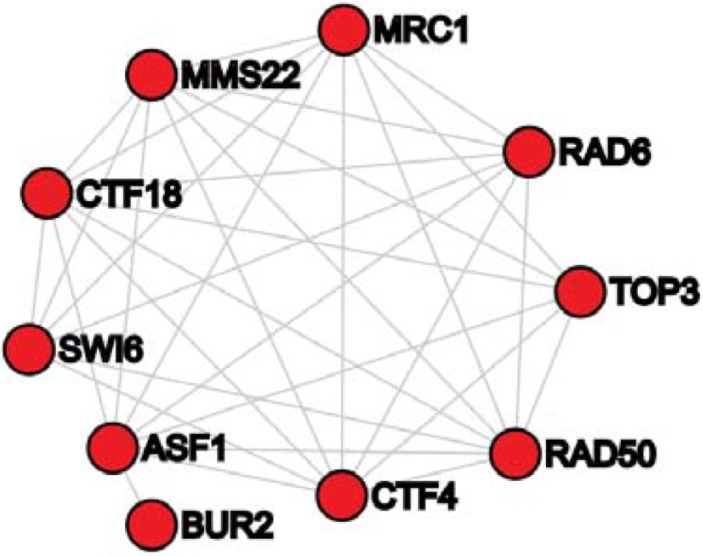
Protein-protein interaction subnetwork of genes in the perturbation module-80.

In another of our perturbation modules, denoted as Module-80 (refer to Fig 7), a literature search showed strong evidence that this perturbation is associated with the function/pathway related to DNA damage as all 10 genes in the Module-80 have been proven to be related to DNA damage. For example, Sharp [30] and Hu [31] et al. found that ASF1 is related to DNA damage. Clausing et al. showed that BUR2 is associated with functions of DNA repair [32]. Fumasoni et al. presented that the DNA damage tolerance relies on CTF4 [33]. Crabbé et al. indicated that CTF18 is essential for DNA damage control [34]. Dovey et al. verified that loss of MMS22 results in the accumulation of spontaneous DNA damage. Xu et al. presented that MRC1 is required for DNA damage checkpoint activation [35]. Karras et al. found the regulation of the RAD6 pathway to DNA damage [36]. Hedglin et al. studied RAD6 activity related to DNA damage tolerance [37]. Chahwan [38] and Roset [39] et al. presented an association of RAD50 to DNA damage. Sidorova and Breeden showed that SWI6 has a function in response to DNA damage [40]. Mankouri [29] and Mohanty [41] et al. presented that TOP3 is related to DNA damage. It is observed that the three genes MMS22, RAD50, and TOP3 of Module-80 are also in Module-30, which mainly regulates the cell cycle. Hence, it is very likely that these 3 genes play multiple roles and genes in Module-80 are in a pathway that regulates a partial function of cell cycle–DNA damage.

We went back to check transcriptomic modules and found that these two perturbation modules and their corresponding transcriptomic modules were functionally coherent. Gene Ontology analysis showed that many genes in the transcriptomic module corresponding to Module-30 are associated with the cell cycle process while many genes in the transcriptomic module corresponding to Module-80 are related to DNA damage, DNA repair, DNA integration etc.

## Conclusions

We used the deep belief network to process the gene expression data and search for transcriptomic modules. One complexity that exists when using deep belief network is the parameter setting, i.e., how to set the proper number of hidden layers and number of nodes in each hidden layer. As there exists no “gold standard”, people usually test different settings manually in order to find a setting that achieves a good performance. In this work, we found that for a given data set, if you gradually increased the number of nodes in each hidden layer, the number of nodes that were actually activated in each hidden layer was bounded by a certain number. In this work, we used those bounds to set the number of nodes in each hidden layer, which resulted in a good performance in terms of finding transcriptomic modules that are biologically meaningful.

The genetic perturbation data obtained from the gene deletions is a valuable resource for studying signaling pathways. The basic idea is that if the deletion of a gene perturbs a signaling pathway, then the expression levels of genes regulated by the pathway will change significantly. By comparing the expression profiles, we could obtain relevant information to decide if the deletions of two individual genes perturb a common signal. However, as 1) there exist some random perturbations, or even just because of that cells may be in the different phases of cell cycle, and 2) a gene/protein, such as CDC42, in a pathway can take roles in other pathways [42]. Hence, besides the genes regulated by the pathway, some other genes can also be differentially expressed in the gene deletion experiments, which causes problems if we are comparing the genome-wide expression profiles. In this work, in order to greatly reduce the above problems, we first found transcriptomic modules such that genes in each module are highly likely to be regulated by a common pathway. We then only compared the expression profiles on genes in transcriptomic modules. Our results showed that utilizing the intrinsic relation between the perturbation of a pathway and the expression changes of genes regulated by the pathway is very helpful for studying the signaling systems.

There exist other data sets that used small molecules or drugs to perturb cell signaling systems and obtained the expression profile changes of cells, such as CMap data [43, 44] and LINCS data [45, 46]. As those expression data were basically obtained from single perturbation, our new computational model can also be applied to those data to find what small molecules or drugs perturb the same pathway. As a result, clinicians have the option to target other genes in a pathway if targeting one gene in this pathway does not work for a patient in targeted therapy.

## Supporting information

S1 Fig A global enrichment analysis of top 20 most enriched GO terms and their matched transcriptomic modules. In order to more easily view the result, we have taken negative log values of enrichment p-values and then normalize them.
(EPS)

S2 Fig A global enrichment analysis of top 20 most enriched GO terms and their matched perturbation modules. In order to more easily view the result, we have taken negative log values of enrichment p-values and then normalize them.
(EPS)

S3 Fig Comparing the GO enrichment analysis of perturbation modules obtained from using binarized fold-change, original fold-change, and normalized fold-change. (EPS)
